# Pervasive Transcription in the Human Genome Exceeds Background Noise

**DOI:** 10.1093/gbe/evag042

**Published:** 2026-02-25

**Authors:** Brett N Adey, Danielle J Maddock, Sylvie Hermann-Le Denmat, Marcel E Dinger, Paul P Gardner, Anthony M Poole, Austen R D Ganley

**Affiliations:** School of Biological Sciences, University of Auckland, Auckland, New Zealand; School of Biological Sciences, University of Auckland, Auckland, New Zealand; School of Biological Sciences, University of Auckland, Auckland, New Zealand; School of Life and Environmental Sciences, Faculty of Science, University of Sydney, Sydney NSW 2006, Australia; Department of Biochemistry, University of Otago, Dunedin, New Zealand; School of Biological Sciences, University of Auckland, Auckland, New Zealand; School of Biological Sciences, University of Auckland, Auckland, New Zealand

**Keywords:** pervasive transcription, background noise, junk DNA, noncoding RNA, deep-learning, random DNA

## Abstract

Large genomes such as the human genome are pervasively transcribed yet encode relatively few unambiguously functional elements. This has led to debate over whether pervasive transcription is indicative of large suites of uncharacterized functional elements or is simply background noise. Here, we used a deep-learning model to estimate background transcription in the human genome as a way of distinguishing between these two hypotheses. We applied the model to randomized (reversed or shuffled) versions of the human genome and found that transcription is predicted to be sparse across all randomization methods, initiating with at least four-fold lower frequencies than in the native human genome. This relatively low level of background transcription from the human genome suggests that most transcription is not a consequence of background noise, thus it requires other explanations. We find that randomizing only interspersed repeats in human genome has little impact on predicted transcription, suggesting that transcription of mobile elements does not explain the excess transcription in the human genome. Instead, most transcriptional events may derive from functional noncoding RNA transcripts, some general requirement for extensive transcription initiation/elongation, and/or mutational biases leading to the frequent appearance of transcription initiation sites by chance.

Significance Our work provides an important step in resolving the long-standing debate over what proportion of the human genome is functional. A key aspect of this debate is whether pervasive transcription provides evidence for the existence of a large suite of uncharacterized functional elements or is the result of background noise. Our major finding is that while there is appreciable background transcription in the human genome, it is much lower than total transcription. We found this by employing machine learning to predict transcription initiation in randomized versions of the human genome as a way of estimating background transcription. We also found the excess transcription is not the result of transposon transcription. Our results indicate that much of the human genome pervasive transcription cannot be attributed to noise. This in turn suggests the high levels of transcription are either beneficial and thus attributable to selection, or result from mutation bias resulting in the creation of transcription initiation sites. Finally, we report a trimodal distribution of distances between transcription initiation sites that is remarkably robust to genome randomization.

## Introduction

A major question in genome biology is the extent to which large genomes like the human genome are made up of nonfunctional “junk” DNA versus as-yet uncharacterized functional “dark” DNA. This question has generated vigorous debate, and a key point of contention is how to interpret observations of pervasive transcription (Eddy 2013; Graur et al. 2013; Kellis et al. 2014; Doolittle and Brunet 2017; Jandura and Krause 2017; Walter 2024). Pervasive transcription refers to the widespread transcription of genomes including the regions that do not harbor any known functional elements (Kapranov et al. 2007). For example, the ENCODE project reported that at least 75% of the human genome is transcribed (Djebali et al. 2012), even though only ∼2% of the genome encodes proteins (Piovesan et al. 2019). One view is that pervasive transcription is just a consequence of background noise—simple transcription initiation requirements result in many nonfunctional transcripts being produced (Struhl 2007; Palazzo and Gregory 2014; Koonin 2016). The other view is that pervasive transcription is a manifestation of the widespread presence of uncharacterized functional elements, particularly noncoding RNA genes, that have important functions in processes such as cell-type-specific regulation (Pennisi 2012; Mattick and Dinger 2013; Jandura and Krause 2017). Although various aspects of transcription have been claimed in support of one view or the other (eg (Deveson et al. 2018; Pertea et al. 2018; Wright et al. 2022; Marasco and Kornblihtt 2023; Walter 2024), the debate remains unresolved.

One approach for determining the extent to which pervasive transcription is a consequence of background noise is to examine the expression profile of “random DNA” to establish the baseline level of transcription (Eddy 2013). If transcription levels in random DNA are similar to transcription from natural genomes, then pervasive transcription is not evidence for function. However, if transcription in the native genome is higher than that observed in random DNA, something other than background noise would be required to explain the excess transcription. A recent study employed this random DNA approach by creating a reversed version of a 100 kb sequence containing the human *HPRT1* gene and introducing it into mouse embryonic stem cells and the yeast *Saccharomyces cerevisiae* (Camellato et al. 2024). Reversing DNA sequences destroys the information encoded in them, thus essentially randomizing the sequences whilst preserving some sequence properties (Deveson et al. 2016). The authors found the reversed sequence was pervasively transcribed in *S. cerevisiae* (Camellato et al. 2024), as has been observed for other heterologous sequences introduced into that species (Zhou et al. 2022; Gvozdenov et al. 2023; Luthra et al. 2024; Meneu et al. 2025). However, they found the reversed sequence was completely transcriptionally silent in mouse (Camellato et al. 2024). While these results suggest that background transcription in mammalian genomes is low, it has faced criticism that a single 100 kb sequence is insufficient in scale to form a true level of baseline transcription, because the transcript distribution is still patchy at the 100 kb scale in the native human genome (Eddy 2024).

Here, we assessed the level of background transcription in the human genome at a genome-wide scale. We did this by applying a deep-learning transcription model, Puffin-D (Dudnyk et al. 2024), to predict transcription initiation across rearranged versions of the entire human genome sequence. We found appreciable levels of background transcription initiation are predicted, but these levels are still much lower than the levels observed in the native genome. We found the level of background transcription depends on the sequence properties of the random DNA and that transcription in the native genome is not driven by transposons. Therefore, we conclude that much of the transcription observed in the human genome cannot be attributed to background noise, suggesting that pervasive transcription results either from selection for transcriptional activity or from mutational bias creating an overrepresentation of transcription initiation sites.

## Results

### Puffin-D Accurately Predicts Lack of Transcription Initiation in Reversed Sequences

We set out to determine the level of background transcription in human random DNA using the recently published transcription initiation predictor, Puffin-D (Dudnyk et al. 2024). We chose Puffin-D, as it is a deep learning model trained on human transcription initiation datasets that predicts transcription initiation at single-nucleotide resolution (Dudnyk et al. 2024). Puffin-D predicts signals for the five TSS detection methods it was trained on (FANTOM CAGE, ENCODE CAGE, ENCODE RAMPAGE, GRO-cap, and PRO-cap). It predicts transcription initiation for both strands across a 100 kb sequence input, thus giving a total of 10 predictions for each base pair in the input sequence.

Puffin-D was shown to have good performance on synthetically constructed random promoter sequences (Dudnyk et al. 2024). However, we wanted to determine whether it can accurately predict transcription initiation of truly out-of-distribution “random” DNA. To do this, we used two reversed sequences from a ∼100 kb region around the human *HPRT1* locus that were created by the only study we are aware of that has measured transcription of long random DNA sequences in mammalian cells (Camellato et al. 2024). Although Puffin-D was trained on human data and the study by Camellato and colleagues was performed in mouse embryonic stem cells, it performed well in predicting transcription initiation in the mouse genome (Dudnyk et al. 2024), and cross-mammalian performance has also been shown for other transcription prediction models (Barbadilla-Martinez et al. 2025). Therefore, we compared the Puffin-D transcription initiation predictions for the two *HPRT1*-derived reversed sequences and the *HPRT1* forward sequence to the experimentally observed mouse GRO-cap data for these sequences (Camellato et al. 2024). As expected, Puffin-D accurately predicted transcription initiation in the forward sequence (Fig. S1 and S2). In contrast, no transcription initiation (at the level of one pseudo-read or more) was predicted for either of the reversed *HPRT1*-derived sequences, including one with all CpG sites removed (Fig. S2). These results mirror the experimental results, which found essentially no transcription of either of these sequences (Camellato et al. 2024). This demonstrates that Puffin-D is capable of accurately predicting transcription initiation for sequences it was not trained on.

### Puffin-D Predicts Relative Low Transcription Initiation Rates in a Reversed Human Genome Compared to the Native Genome

**Fig. 1.**
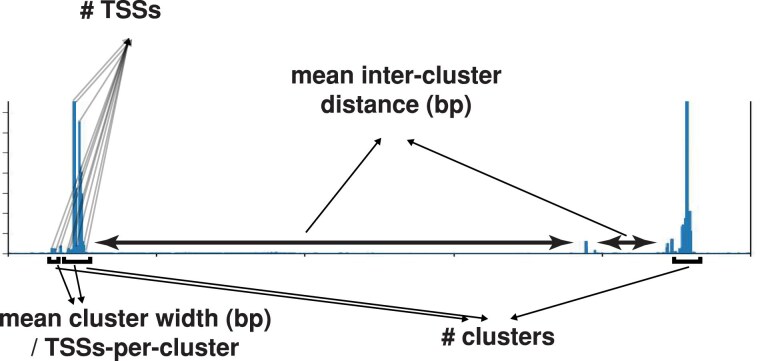
Transcription initiation metrics. The transcription initiation metrics used in this study are displayed around a raw Puffin-D transcription initiation prediction output plot. Individual vertical blue bars are TSS predictions; the *x*-axis is equivalent to one 100 kb input sequence. Transcription initiation clusters, which are collections of TSSs where the maximum distance between any two TSSs is 25 bp, are indicated, and a given cluster may contain one or more TSSs, thus allowing us to calculate cluster width (in bp) and number of TSSs in a cluster.

We next set out to determine how much transcription initiation Puffin-D predicts from *in silico* randomized versions of the human genome. We initially adopted the approach of reversing native sequences used by Camellato and colleagues (Camellato et al. 2024), creating a random genome by reversing the whole human T2T genome (Nurk et al. 2022). We then used Puffin-D to predict transcription initiation in both the “forward” (native) and “reversed” versions. We calculated several transcription initiation metrics (Fig. 1), then compared the results to those from an ENCODE experimental GRO-cap dataset (ENCFF799JUK).

Puffin-D predicted a similar number of transcription start sites (TSSs) in the forward sequence to that observed in the experimental GRO-cap data (Table 1). However, when we applied Puffin-D to the reversed T2T sequence, we found the number of predicted TSSs is only ∼25% of the number predicted for the forward genome and observed in the experimental data. We wondered if transcription initiation was being predicted more stochastically in the reversed genome, in contrast for the tendency of real TSSs to form TSS clusters (Carninci et al. 2005; Zhao et al. 2011; Bernardini et al. 2023). However, clustering TSSs that are 25 bp or less apart (Policastro and Zentner 2021) surprisingly showed the opposite pattern: clustering is weaker in the forward than the reversed genome and weaker again in the experimental data (Table 1), as determined both by how wide clusters are on average and how many TSSs occur on average within a cluster (Fig. 1). This suggests that Puffin-D recognizes fewer regions of transcription initiation but populates them with a higher density of TSSs than is observed in vivo. It is possible this difference is because the lymphoblastoid B cell-specific nature of the experimental GRO-cap dataset differs from the non-cell type specific predictions made by Puffin-D, although it could be a consequence of Puffin-D not having fully learned transcription initiation rules. Regardless, the sparsity of transcription initiation predicted in the reversed genome results in far higher average inter-cluster distances than those seen for the forward and experimental GRO-cap datasets (Table 1). Together, these results suggest that reversing the human genome results in a dramatic reduction of transcription initiation, although not a complete abolition. Thus, background transcription initiation appears to occur in randomized sequences, but at a level too low to account for most pervasive transcription observed in the native human genome.

**Table 1:** Effects of randomizing the human genome on transcription initiation

Dataset	Number of TSSs	Number of TSS clusters^a^	Mean cluster width^b^	Mean number of TSSs per cluster	Mean inter-TSS distance	Mean inter-cluster distance
Experimental GRO-cap	2,218,983^c^	741,856	11.8	2.99	1,358	4,051
T2T forward	2,258,412	287,993	26.5	7.84	1,377	10,772
T2T reversed	522,771	47,125	44.5	11.09	5,924	65,703
Local dinucleotide shuffled	444,570	67,639	25.6	6.57	6,978	45,853
Global dinucleotide shuffled	17,272	6,338	11.8	2.73	175,000	478,038
Local mononucleotide shuffled	8,498	2,332	14.5	3.64	306,682	1,125,994
Global mononucleotide shuffled	101	52	5.3	1.94	5,721,320	13,731,162
Reversed repeats^d^	1,490,275	267,250	20.8	5.58	2,087	11,616
Reversed non-repeats^e^	600,400	80,708	26.0	7.44	5,170	38,447
Repeats globally shuffled	963,766	129,563	25.7	7.44	3,225	23,971
Non-repeats globally shuffled	1,415,927	182,790	26.4	7.75	2,194	16,974
Forward holdout chromosomes^f^	218,958	31,144	24.2	7.03	1,969	13,823

^a^Clusters are groups of TSSs where the maximum distance between any two adjacent TSSs is 25 bp.

^b^Average span of TSS clusters in bp.

^c^Experimental GRO-cap TSS counts are not directly comparable to *in silico* genome data because different reference genomes were used, but the difference is negligible (see **Materials and Methods** for details).

^d^All repeats in the T2T genome are reversed; other sequences are unchanged.

^e^All repeats in the T2T genome are unchanged; other sequences are reversed.

^f^Predictions for just the human chromosomes (8, 9, and 10) that Puffin-D was not trained on.

### Preservation of Local Dinucleotide Structure Results in Higher Predicted Levels of Transcription Initiation

A previous deep-learning study found that local dinucleotide content strongly impacted predictions of chromatin state, with local preservation of dinucleotide content predicted to have higher levels of active chromatin than when global dinucleotide shuffling or mononucleotide shuffling were employed (Luthra et al. 2024). To test whether Puffin-D also predicts an effect of dinucleotide composition on transcription initiation, we created shuffled versions of the human T2T genome with dinucleotide and mononucleotide shuffling both locally and globally. We found Puffin-D predicted less transcription initiation in all four datasets compared to the native genome. We found progressive loss in predicted transcription initiation as shuffling became more aggressive, with mononucleotide global shuffling all but abolishing predicted transcription initiation sites (Table 1). These results support the contention of Luthra and colleagues that dinucleotide composition is an important part of the information content of the human genome (Luthra et al. 2024). Interestingly, the local dinucleotide shuffled and reversed datasets show similar TSS counts (444,570 vs. 522,771), suggesting that the level of transcription initiation in the reversed sequence may be due to some local sequence composition being preserved. Taken together, these results suggest that randomizing the human genome produces a range of transcription initiation levels, ranging from almost no initiation through to ∼25% of the level observed in the native human genome, depending on the nature of the randomization. However, in all cases, the predicted level of transcription initiation is far lower than that observed in the native genome.

### Transcription Initiation Above Background Occurs Predominantly in Non-repeat Regions of the Native Human Genome

Our results suggest that the background level of transcription initiation is lower than that observed in the native human genome, implying that something beyond background transcription is required to explain observed human transcription. Given that transposable elements (TEs) have been reported to contribute substantially to TSSs in humans (Faulkner et al. 2009; Francescatto et al. 2017; Fueyo et al. 2022), one possible explanation is that pervasive transcription in the human genome is the consequence of transcription from TEs. To investigate how much transcription in the native genome comes from TEs, we divided the human T2T genome into repeat regions (the majority of which are TEs) and non-repeat regions. We then used global mononucleotide shuffling to randomize the repeat regions, and the non-repeat regions as controls, and predicted transcription initiation in these shuffled genomes with Puffin-D.

**Fig. 2.**
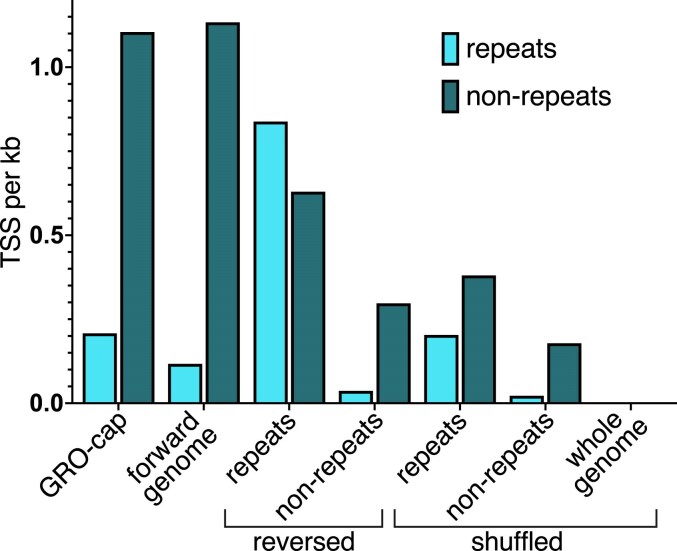
Effects of randomizing the repeat and non-repeat regions of the T2T human genome on transcription initiation frequency in these regions. The numbers of TSSs per kb in the repeat and non-repeat regions predicted by Puffin-D for versions of the human genome with repeat regions randomized and with non-repeat regions randomized (by sequence reversal and shuffling) are plotted. For comparison, TSS per kb is also plotted for the experimental GRO-cap data, and for Puffin-D predictions of the forward whole human genome and the whole shuffled genome. Shuffling is all mononucleotide global.

We found that TSSs are depleted in both experimental GRO-cap and Puffin-D predicted forward T2T genome repeat regions in comparison to the non-repeat regions, with only ∼6% to 10% of TSSs occurring within repeats despite them occupying 40.3% of the genome (Table 2). Interestingly, shuffling the repeats actually increases predicted transcription initiation frequency in repeats (Fig. 2), although it reduces predicted initiation from non-repeat regions. Moreover, repeats contribute to transcription in non-repeat regions as shown by the further decrease in predicted non-repeat transcription when the entire genome is shuffled compared to when only the non-repeat regions are shuffled (Fig. 2). This suggests the transcriptional machinery does not treat repeat and non-repeat regions independently of each other, as might be expected given their interspersion. Similar results were observed when repeats were reversed rather than shuffled, although, surprisingly, when repeat regions are reversed, predicted transcription initiation in these regions increases in contrast with the decreases observed in the non-repeat regions as expected (Fig. 2). These results provide no support for the idea that a substantial fraction of pervasive transcription in the human genome is TE transcription.

**Table 2:** Effects of shuffling the repeat and non-repeat regions of the human genome on transcription initiation

Dataset	TSSs within repeats	TSSs within non-repeats	Repeat (% total)^a^	Non-repeat (% total)	Total TSS count	TSS per repeat length	TSS per non-repeat length
Experimental GRO-cap	216,778	2,000,498	9.8	90.2	2,217,276	0.208^b^	1.105
Forward^c^	147,285	2,111,127	6.5	93.5	2,258,412	0.117	1.134
Repeat regions shuffled^d^	255,512	708,254	26.5	73.5	963,766	0.204	0.380
Non-repeat regions shuffled	27,759	332,784	7.7	92.3	360,543	0.022	0.179
Whole genome shuffled	73	28	72.3	27.7	101	<0.001	<0.001

^a^Repeat and non-repeat results were calculated from T2T genome annotations. The GRO-cap data come from alignment to the GRCh38 genome, so T2T annotations were lifted over for consistency, resulting in different numbers to Table 1 (see **Methods and Materials** for more information).

^b^Repeat and non-repeat lengths for the GRO-cap data were calculated from total repeat/non-repeat lengths in the GRCh38 genome rather than the T2T genome, as these data were aligned to GRCh38.

^c^Results are all Puffin-D predictions except the GRO-cap data.

^d^All shuffles are mononucleotide global shuffles of the repeat regions only, the non-repeat regions only, or the whole genome (which comes from Table 1).

### Distances Between TSSs Show a Trimodal Distribution

**Fig. 3.**
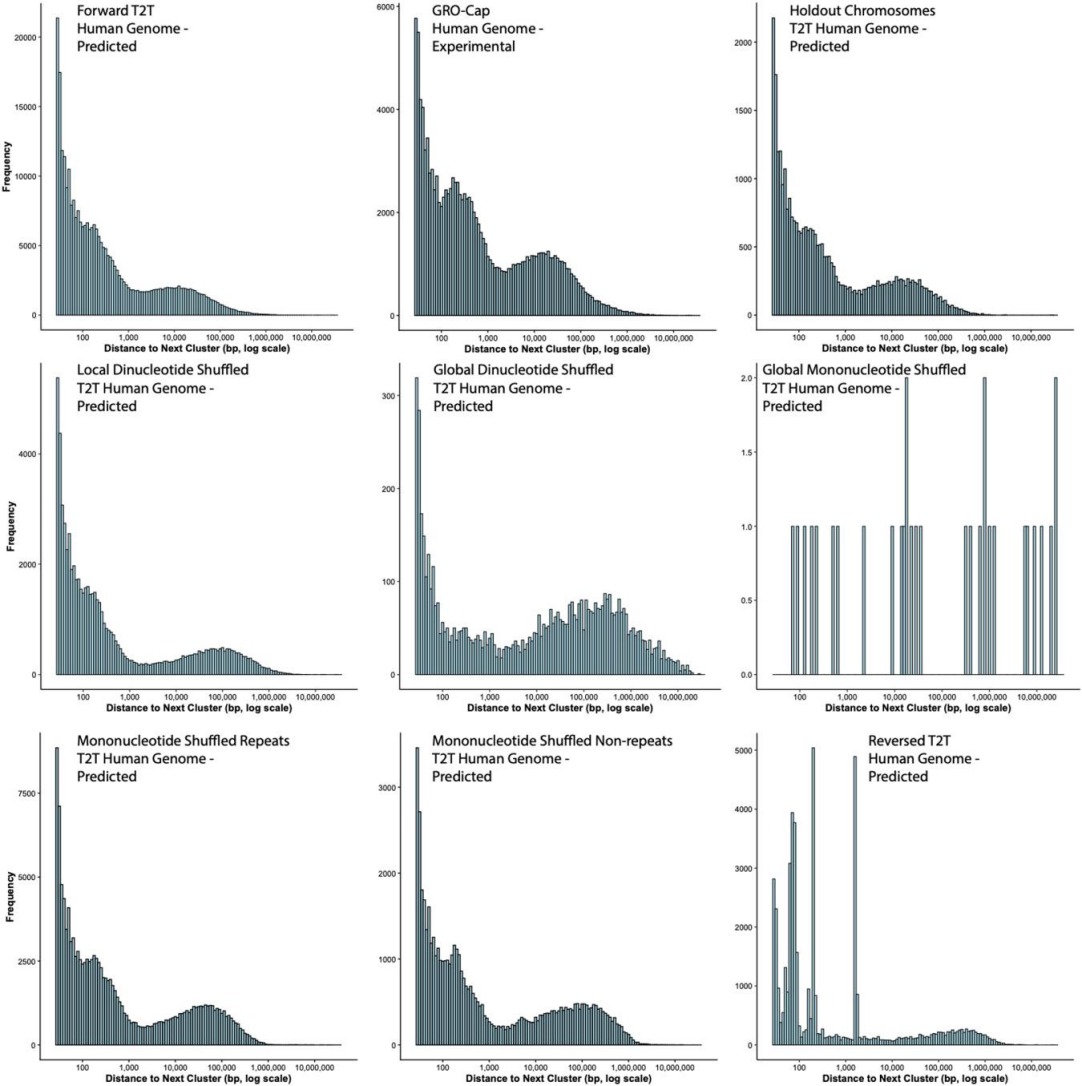
Distances between TSS clusters are trimodally distributed in the native human genome and several randomized genomes. Histograms of distances between TSS clusters from the data in Table 1 are plotted for experimental GRO-Cap data, the predicted forward human genome, predicted forward human chromosomes (8, 9, and 10) not in the Puffin-D training set, local and global dinucleotide shuffled human genomes, the global mononucleotide shuffled human genome, global mononucleotide shuffling of the repeat and non-repeat regions of the human genome, and the reversed human genome, as indicated. Inter-cluster distances are much longer (note the *x*-axis is log scale) as initiation frequency becomes sparser (indicated by the *y*-axis scale). A threshold of six reads to call a TSS was used for the experimental GRO-cap data to make the values comparable to the Puffin-D predicted values. See also Fig. S3.

Finally, we investigated how the spatial distribution of TSSs varies in response to the various genome randomizations we conducted. To do this, we plotted histograms of the distance from each TSS cluster (using the 25 bp clustering threshold) to its next TSS cluster neighbor in the genome. We found that predicted inter-cluster distances in the forward genome show a trimodal distribution of short (<100 bp), medium (centered around ∼300 bp), and long (centered around ∼15,000 bp) distances (Fig. 3).

The trimodal distribution of inter-TSS cluster distances was not initially evident in the experimental GRO-cap data (Fig. S3), but read count magnitudes are greater in this dataset than the pseudo-read counts in the Puffin-D forward prediction (17,880,897 reads vs. 10,914,237 pseudo-reads, respectively). Pseudo-read count magnitudes are arbitrary, as they presumably derive from the read depths of the datasets used to train Puffin-D, so we examined whether this “excess” read depth in the experimental GRO-cap data masks a trimodal distribution. To do this, we enforced different thresholds for the number of reads needed for a site to be called a TSS. As this threshold increases, a trimodal inter-cluster distance distribution very similar to that seen for the predicted forward genome becomes clearly evident in the experimental GRO-cap data (Fig. 3; see also animated GIF on Figshare: https://doi.org/10.6084/m9.figshare.28405775).

The lack of a trimodal distribution when including all GRO-cap reads results from interspersion of inter-TSS distances between the two right-most peaks of the inter-TSS distance distribution when fewer reads are required to call a TSS (fewer required reads is the equivalent of increasing read depth). This suggests that either there are weak TSSs located between the stronger TSSs (that comprise the peaks of the trimodal distribution), or there is a low level of stochastic transcription initiation that is detected if GRO-cap read depth is high enough. To discriminate between these possibilities, we looked at how TSS cluster statistics varied when the threshold read number was changed, based on the premise that TSS cluster metrics will differ for TSSs that appear because of noise. We found that mean TSS cluster size and mean number of TSSs within a cluster are both remarkably robust to changing the threshold (Table S1). Thus, TSSs observed with the low read threshold are probably weak but *bona fide* TSSs, as they have similar clustering metrics to strong TSSs. These results reveal that the distribution of inter-TSS distances is not random for strong TSSs, and possibly also for weak TSSs.

To test whether the ability of Puffin-D to predict this trimodal pattern of inter-TSS cluster distances is a consequence of data leakage from Puffin-D's training set, we plotted the distribution of these distances for the chromosomes (8, 9, and 10) it was not trained on. A very similar trimodal distribution was observed for these three chromosomes (Fig. 3). Thus, the fact that Puffin-D predicts the same distribution of inter-TSS cluster distances as is observed in vivo provides additional support for its ability to accurately predict transcription initiation.

Finally, we were interested to see whether the trimodal inter-TSS cluster distance distribution persists when the genome is randomized. We found the trimodal pattern in most of the shuffled genomes, with the right-most peak drifting to the right (greater inter-cluster distances) as the total number of TSSs declines (Fig. 3). There are two exceptions where the trimodal pattern is lost. The first is the global mononucleotide shuffled genome, which has very few TSSs. The second is the reversed genome (Fig. 3). This is surprising because the number of TSSs is much higher than some shuffled genomes that retain the trimodal distribution (Table 1), and the pattern is still observed when just the repeat or non-repeat regions are reversed (Fig. S3). Thus, the trimodal distribution of inter-TSS cluster distances is robust to most, but not all, genome randomizations.

## Discussion

Here, we show that Puffin-D predicts appreciable levels of background transcription initiation in several randomized versions of the human genome, but in all cases, the initiation levels are much lower than that observed in the native genome. These results suggest that the majority of observed transcription in the native human genome is not readily attributable to background noise, so other explanations for this excess of native genome transcription are required. Our results differ quantitatively to the findings of Camellato and colleagues, who found essentially no background transcription in a reversed DNA sequence (Camellato et al. 2024). This suggests the critique that the length of DNA they analyzed is too short to be extrapolated genome-wide (Eddy 2024) may be valid. However, our results agree qualitatively with theirs, collectively supporting the conclusion that observed transcription from the native human genome is higher than background levels. Our results also differ from a recent study that used deep learning to predict chromatin structure of random DNA in the human genome, as that found predicted chromatin patterns to be similar in native and random DNA (Luthra et al. 2024). We think the discrepancy arises because chromatin profiles are partly a consequence of genomic activities such as transcription (Millan-Zambrano et al. 2022) and that introduced DNA must establish some sort of chromatin structure, making it less predictable solely from DNA sequence than transcription initiation.

The conclusion that much of the transcription observed in the human genome cannot be explained by background noise requires alternative explanations for the excess transcription. We found no support for one possible explanation: that excess transcription is a consequence of selection for TE transcription as part of their selfish lifecycle (Oomen et al. 2025). We conclude this because transcription from TEs is a minority of total transcription, and because randomizing the repeat regions of the human genome does not substantially reduce the number of predicted TSSs. In contrast, our results suggest that overall transcription initiation within repeat regions occurs at background levels, as we found the level of transcription initiation in repeat regions of the native genome is comparable to that seen when the genome is randomized.

Another possible explanation for why transcription levels are above background in the human genome is that transcription has been selected for because it provides benefits to the host. We think there are three main, non-mutually exclusive possibilities for what selection is acting on. The first is selection for a large suite of uncharacterized noncoding RNAs whose transcripts are functional, providing benefits to the host (Mattick and Dinger 2013; Jandura and Krause 2017). The growing list of functions ascribed to noncoding RNAs (Editorial 2022) supports this hypothesis (Walter 2024). However, this explanation is hard to reconcile with observations that most of these elements do not show DNA sequence conservation (Christmas et al. 2023) and that some noncoding RNAs do not appear to encode functional transcripts (Engreitz et al. 2016; Hoeppner et al. 2018; Palazzo and Koonin 2020). Therefore, it is possible that the process of transcription, rather than production of specific transcripts, has been selected for, for example, by displacing proteins bound to DNA to aid in regulation (Kobayashi and Ganley 2005; Berretta and Morillon 2009; Kaikkonen and Adelman 2018). The third possibility is that a large proportion of transcription initiation in the human genome derives from enhancer elements, enabling them to direct expression (Tippens et al. 2020) of a more limited set of transcripts that have one or both of the properties outlined above. The latter two possibilities are consistent with the promoters of noncoding RNAs showing higher conservation than the transcripts they make (Engreitz et al. 2016). However, all these possible benefits of transcription require sufficient selective strength in the human population for the TSSs to have been selected for, something that is debated (Graur 2017).

One possible explanation for observed levels of transcription initiation in the human genome being higher than background does not require selection: mutation bias. Under this scenario, biases in the mutations that arise in the human genome generate sequences whose compositions are more likely by chance to serve as transcription initiation recognition sites than truly random sequences. This explanation is consistent with our observation that predicted TSS activity largely correlates with the level of genome randomization. It is also consistent with a previous report that local dinucleotide shuffling preserved a greater level of predicted active chromatin structure than global shuffling methods (Luthra et al. 2024). Conversely, the most extreme mutation bias in the human genome is against CG dinucleotides, and it was previously shown experimentally that removing CG dinucleotides from a reversed sequence did not increase transcription initiation (Camellato et al. 2024). What could lead to mutation bias producing an overrepresentation of transcription initiation sites? One possibility is the bias mitigates the deleterious consequences of mutation on transcription—ie mutations are less likely to disrupt transcription initiation. Another possibility is that by creating a high level of background transcription, mutation bias provides the conditions where de novo noncoding RNAs/genes can more readily evolve—an evolution of evolvability scenario—although it may be unlikely that selection acts in this way (Poole et al. 2003).

Our results suggest that while not explaining all observed transcription, there is probably an appreciable level of background transcription from the human genome. This background transcription must result from chance occurrences of motifs that are predicted to initiate transcription, consistent with the idea that recognition of relatively small sequences by transcription factors with imperfect binding specificities drives transcription (Jolma et al. 2013; Lambert et al. 2018; Sahu et al. 2022; Dudnyk et al. 2024). Robust transcription is suggested to depend on clusters of degenerate TF binding sites (Khetan et al. 2025; Xie et al. 2025). Here, we did not assess transcription initiation magnitude (how much initiation is predicted from a site), as Puffin-D is not designed to make magnitude predictions (Dudnyk et al. 2024). However, if robust transcription is dependent on the degenerate TF binding site clusters observed at native human initiation sites, transcription initiation driven by TF binding sites appearing by chance is expected to be weaker. Synthesizing some of the randomized sequence TSSs predicted in this study and experimentally measuring their transcription levels is one way this could be tested. Finally, machine-learning approaches may be able to identify patterns, such as transcription initiation rate, enhancer activity, and TF binding site cluster density, that distinguish background TSSs from those that have been selected to enable identification of functional TSSs.

Our results revealed a trimodal distribution of inter-TSS cluster distances, which as far as we know has not been previously reported. We suspect the short distances (left-most peak) reflect true transcriptional initiation clusters—ie sites that are responsible for initiating transcription of the same gene/transcriptional element (Carninci et al. 2005; Zhao et al. 2011; Bernardini et al. 2023). If so, our results suggest that transcription initiation clusters are typically up to ∼100 bp in length, in agreement with experimental observations of transcription initiation cluster sizes (Zhao et al. 2011; Bernardini et al. 2023). We suspect the longest distance peak (right-most peak) represents intergenic distances (ie the distance between the transcription initiation sites of one gene and its neighbor). The nature of the intermediate peak, though, is less clear. Although TEs are typically around this length (Cordaux and Batzer 2009; Wells and Feschotte 2020; Luqman-Fatah et al. 2024), they are unlikely to be responsible for this peak, as the trimodal pattern persists even when repeats are randomized. It is also curious that the trimodal distribution is robust to most genome sequence randomization, although sequence reversal essentially abolishes the pattern. This may suggest that dinucleotide content is somehow important for the trimodal pattern, but it is unclear why this should be.

In summary, we found that transcription initiates in *in silico* randomized human genomes at only about one-quarter of the level observed in the native human genome. This disparity is unlikely to be explained solely by the presence of genes in the native genome, as the number of TSS clusters in the T2T forward genome (742,037 clusters with a maximum cluster gap of 25 bp) is vastly greater than the number of genes. On the basis of these results, we conclude that the level of background transcription initiation activity in the human genome is relatively low and does not account for the majority of the transcription in the human genome. We found that TE transcription is unlikely to explain excess transcription in the human genome. Instead, we suggest two non-mutually exclusive explanations for the majority of transcription in the human genome. The first is that this transcription results from selection, ie there are a substantial number of uncharacterized functional noncoding transcripts and/or the process of transcription per se potentially provides some selective benefit, such as displacing bound proteins. The other is that mutational bias leads to overrepresentation of sequences that are capable of initiating transcription. These conclusions are predicated on the ability of Puffin-D to predict transcription initiation in non-native sequences, which we base on its ability to accurately predict the lack of transcription in the reversed *HPRT1* sequences and to predict a previously unrecognized trimodal distribution of inter-TSS cluster distances. However, further experimental tests of the transcriptional activity of random DNA in human cells are required to confirm Puffin-D's accuracy in predicting out-of-distribution sequences, and such experiments should ideally be done in human cells, rather than mouse (Camellato et al. 2024). In addition, Puffin-D predicts transcription following training on numerous different cell types, therefore its predictions might not relate to any particular cell type, including the lymphoblastoid B cell that the experimental GRO-cap ENCODE data used here were derived from. Thus, it will be valuable to determine, either through experiments or by developing cell-type specific prediction models, how much cell type affects the transcription of random DNA. Nevertheless, our results indicate that the level of transcription in the human genome is much higher than the baseline expected from noise. Therefore, urgent investigation is required to determine whether this excess transcription is functional and if so in what ways.

## Materials and Methods

### TSS Prediction Using Puffin-D

Puffin-D was obtained from the *jzhoulab* GitHub page (https://github.com/jzhoulab/puffin; downloaded 08/11/24) and was used to predict transcription initiation by running Python (v. 3.9.23) with the sequence function in CPU mode as follows:

*python puffin_D.py sequence file.fasta*

Puffin-D outputs continuous read count predictions in the form log10(s + 1) for each nucleotide position in the input sequence. To compare Puffin-D predictions to experimental read counts for the *HPRT1* data, and the total number of reads predicted by Puffin-D to the number in the experimental GRO-cap dataset, we converted these log10(s + 1) values into what we call pseudo-read counts by 10^[Puffin-D Prediction]^-1. All values greater than one were rounded to the nearest pseudo-read, and values less than 1 were set to zero.

### Nucleotide Sequence Generation

We obtained the 100,667 bp human *HPRT1* sequence from Camellato et al. (2024), truncated 667 bp from the 3′ end to meet the 100 kb length requirement for Puffin-D, and reversed this with SeqKit to obtain *HPRT1R*. We obtained the *HPRT1RNoCpG* sequence, which is a version of *HPRT1R* with all CpG sites removed, from Camellato et al. (2024). This sequence was 95,067 bp, so to meet the 100 kb length requirement of Puffin-D we acquired the intergenic sequences flanking this sequence from the UCSC genome browser and appended them to make a 100 kb sequence.

The T2T genome assembly fasta file (Nurk et al. 2022), *GCF_009914755.1_T2T-CHM13v2.0_genomic.fna.gz*, was retrieved from NCBI. To meet the 100 kb length requirement of Puffin-D, we divided the assembly into single-line 100 kb units using seqkit (v. v2.10.1) as follows:

*seqkit sliding -s 100000 -W 100000 | seqkit seq -w 0*

We then separated these by chromosome using awk. To create the reversed T2T human genome sequence, each 100 kb unit was reversed with *seqkit seq -r.*

#### T2T Genome Shuffling

The human T2T genome was randomized using local dinucleotide, global dinucleotide, local mononucleotide, and global mononucleotide random shuffles on the by-chromosome single-line 100 kb fasta files from above using Biasaway (v. 3.3.0) following the method used in Luthra et al. (2024). This was achieved as follows:

Local dinucleotide and mononucleotide shuffling (implemented within 100 bp sliding windows):

*biasaway w -k 2 -w 100 -s 50* and *biasaway w -k 1 -w 100 -s 50*, respectively

Global dinucleotide and mononucleotide shuffling:

*biasaway k -k 2* and *biasaway k -k 1*, respectively

To return the sequences to the single-line fasta file format required for Puffin-D input and to update the headers, *seqkit replace* and *seq -w 100000* were performed for all shuffles.

#### T2T Genome Repeat-based Reversal and Shuffling

The T2T genome is softmasked for repeats (repeats set to lower case). To create repeat-based randomized genome sequences, we used the Biopython, re, io, and collections modules in Python. Each contiguous run of upper (for the non-repeat shuffle) or lowercase (for the repeat shuffle) nucleotides was extracted with a regular expression, independently shuffled, and reinserted into the same position using Python's double-ended *queue, deque*. Shuffling was performed using global mononucleotide shuffling with Biasaway as above.

The repeat-reversed and non-repeat-reversed versions of the T2T genome were generated similarly, except by reversing lower or uppercase contiguous sequence runs using slice syntax in Python. Summary information for the repeat and non-repeat analyses was generated in R (v. 4.4.2) using the data.table, IRanges, GenomicRanges, and tools packages. Plots within each class (“repeat” or “non-repeat” region) were also made in R with the tidyverse packages dplyr, readr, tidyr, and ggplot2 (including scales).

### Transcription Initiation Metrics

#### Transcription Initiation Metrics

Transcription initiation metrics (number of TSSs; number of TSS clusters; mean TSS cluster width; mean number of TSSs per cluster; mean inter-TSS distance; and mean inter-TSS cluster distance) were calculated using a custom R script *TSS_Distance_Analysis.R* for all datasets except the experimental GRO-cap dataset, for which we used the custom script *Grocap_TSS_Distance_Analysis.R* (scripts are available on Github).

#### Experimental GRO-Cap Data

An experimental GRO-cap dataset from a human lymphoblastoid B cell (GM12878) aligned to the GRCh38 reference genome was downloaded from ENCODE (ENCFF799JUK). The sense strand was converted into bedgraph format with the UCSC *bigWigToBedGraph* tool (v. 448) and used for subsequent comparisons.

The *HPRT1* region (ChrX:134429875-134529874) was extracted from the experimental GRO-cap data bedgraph file using awk and combined with the Puffin-D predictions of the various *HPRT1* sequences using using dplyr bind_rows in R before plotting the read counts using ggplot2.

To standardize the coordinates of the experimental GRO-cap data, which were mapped to the GRCh38 reference genome, to those of the Puffin-D predictions for the whole-genome sequence versions, which are based on the T2T genome, we converted all experimental GRO-cap reads that map to GRCh38 to the equivalent positions in the T2T reference genome using LiftOver (https://hgdownload.cse.ucsc.edu/admin/exe/linux.x86_64/liftOver; downloaded 28/06/25). 2,217,276 of 2,218,983 positions were successfully lifted over to T2T reference coordinate space, while 1,707 positions were unmapped.

### Comparing Repeat and Non-repeat Regions

To compare TSS count within the repeat/non-repeat classes, we first created.bed files with the repeat and non-repeat region coordinates from the T2T genome softmasking using regular expressions. Next, because the experimental GRO-cap data were aligned to GRCh38 coordinates, while the other datasets were based on the T2T genome coordinates, we ported the T2T repeat/non-repeat masks to GRCh38 reference genome using LiftOver.

To standardize the number of predicted TSSs by class length (repeat/non-repeat sequence length), we first counted the lengths of the repeat and non-repeat regions within the GRCh38 genome. We then used these class lengths (GRCh38 lengths for the experimental GRO-cap dataset and T2T lengths for the other datasets) as denominators to calculate TSS per class length from the TSS counts.

### TSS Distance Analyses

All TSS distance analyses and plotting were conducted in R, with the future.apply package used for parallelization and the IRanges package used for TSS distance calculation. Data.table and tidyverse packages dplyr, readr, forcats, and ggplot2 (including scales and viridis) were used for data manipulation and plotting.

Clustering of TSS into TSS clusters was achieved by collecting intervals between TSSs (integer intervals) within the IRanges package, then merging these intervals into clusters by adjusting the “Max Gap” parameter, which defines the maximum number of positions without a TSS signal allowed between TSSs for those TSSs to form part of the same cluster.

To assess whether read depth was masking a tri-modal distribution in the experimental GRO-cap data, increasing values of a “threshold” parameter—the minimum number of reads required to call a TSS—were applied before calculating inter-initiation site distances using IRanges as above.

## Supplementary Material

evag042_Supplementary_Data

## Data Availability

For additional information and figures, please see the Supplementary Figures and Supplementary Tables. The following files can be found on Figshare (https://doi.org/10.6084/m9.figshare.28405775.): Human T2T forward sequences (as*.fasta* files split by chromosome in *Fastas/T2T_fastas_by_chr*). *HPRT1* sequences and a 100 kb human genome sequence selected from a holdout chromosome of Puffin-D (as 100 kb*.fasta* files in the *Fastas* folder). Raw Puffin-D output results for the 100 kb holdout sequence and the various *HPRT1* sequences (*Puffin-D Numpy Arrays* folder). Experimental GRO-cap data variants: Experimental GRO-cap data of *HPRT1* region only (*enbw_plus_HPRT1.bedgraph*); experimental GRO-cap data formatted to one nucleotide per row (*enbw_plus_perbp.bedgraph)*; and experimental GRO-cap data lifted to the T2T genome (*GROcap_chm13_nc_sorted.bed).* Liftover files for the experimental GRO-cap data and repeat masks. A list of datasets and corresponding GRO-cap TSS position bed files (*datasets_positions.tsv*) Movie file showing how the inter-cluster distance distribution of the experimental GRO-cap dataset changes with differing read threshold values (*Inter-cluster distance distribution across read thresholds movie*). All scripts used for this work can be found on Github (https://github.com/BrettAdey/TSS-Prediction-Project).
