# Supplementary material for: Pervasive Transcription in the Human Genome Exceeds Background Noise
Source: Genome Biol Evol. 2026 Feb 25;18(5):evag042. doi: 10.1093/gbe/evag042 (PMC13171451; doi:10.1093/gbe/evag042)
Supplement: evag042_Supplementary_Data [file evag042_supplementary_data.pdf]

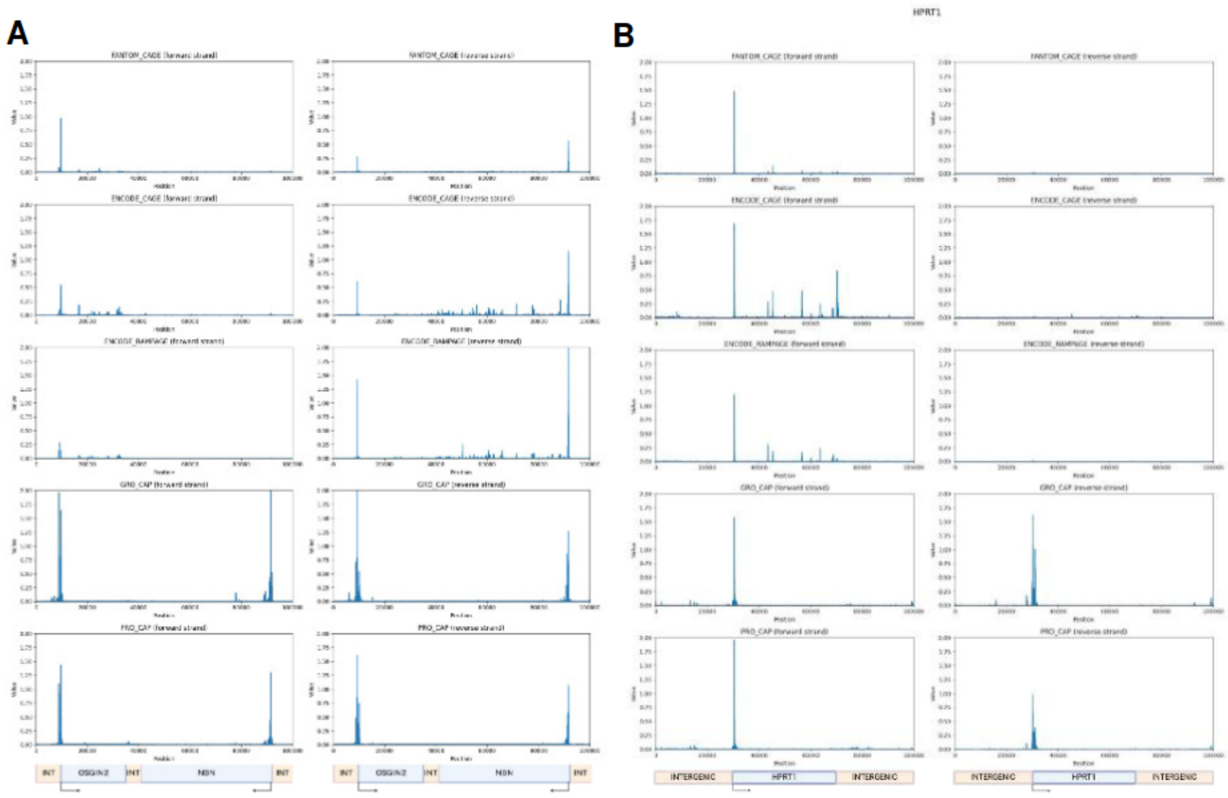

Figure S1. Puffin-D accurately predicts transcription initiation in known sequences. Transcription initiation predictions of a region of the human genome known to be transcribed are shown for the forward (left column) and reverse (right column) strands for the five different transcription initiation data types predicted by Puffin-D (FANTOM CAGE, ENCODE CAGE, RAMPAGE, GRO-Cap, PRO-Cap). The genomic region is located in one of the holdout chromosomes that Puffin-D was not trained on (Chr8: 89,892,879 - 89,992,878). **B.** Same as **A**, except the transcription initiation predictions are for the *HPRT1* forward sequence from Camellato, et al. (2024). Below each panel is a visualisation of the gene organization of the region, indicating genes (blue boxes; arrows show orientation of transcription) and intergenic regions (orange boxes). Puffin-D predicts the bi-directional nature of transcription initiation that is seen for experimental measures of nascent transcription (Core, et al. 2008; Seila, et al. 2008), particularly for the two run-on based transcription initiation assay methods (GRO-cap and PRO-cap). Predictions were broadly similar across transcription initiation assays, therefore to simplify analyses we opted to focus on a single, nascent transcription initiation assay type, GRO-cap, which has the advantage of transcription initiation measures not being confounded by rates of transcript degradation or decay (Core, et al. 2008).

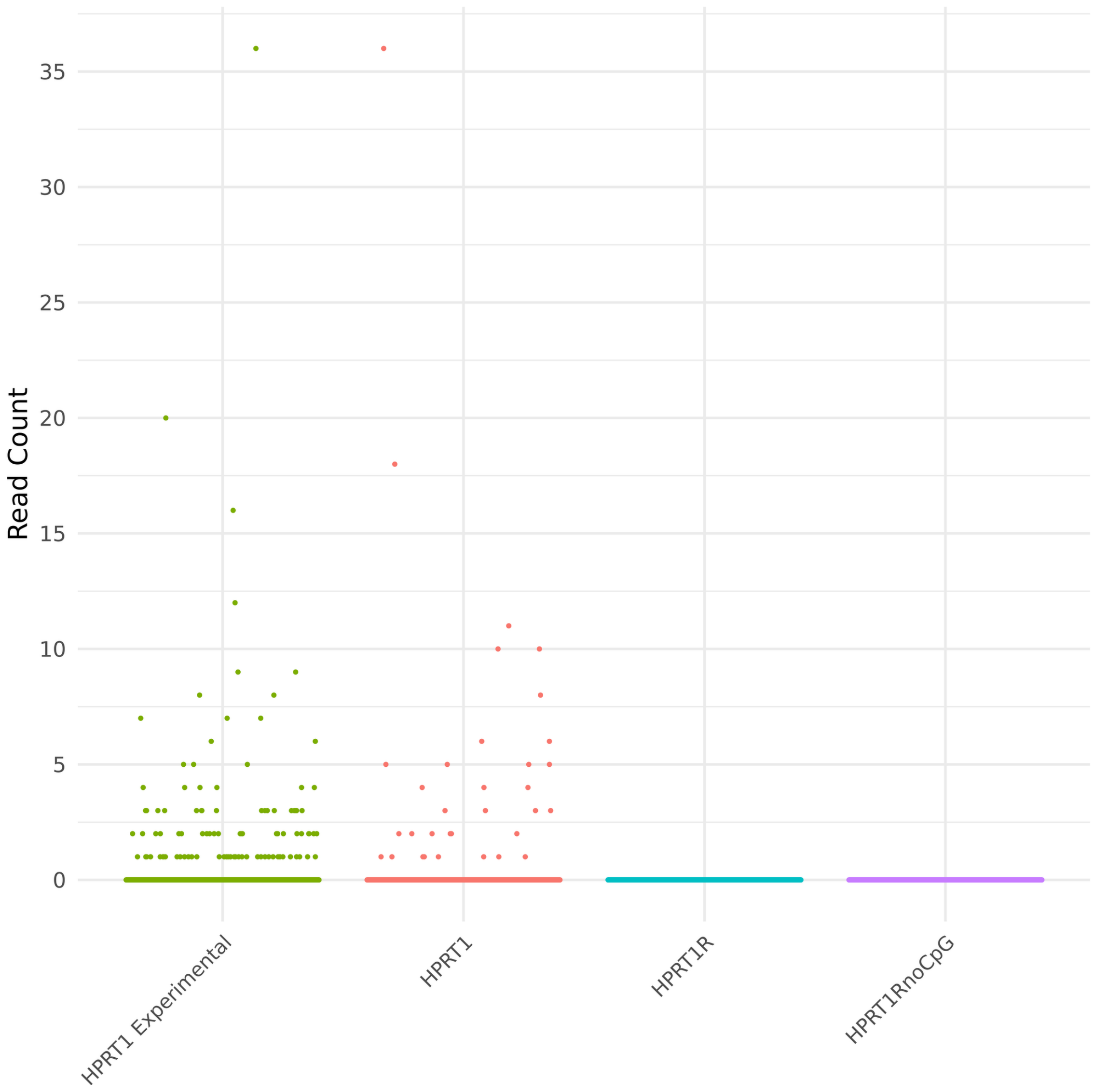

Figure S2. Puffin-D correctly predicts lack of transcription initiation in reversed *HPRT1* sequences. Puffin-D predicts transcription initiation in the forward *HPRT1* sequence similar to that observed experimentally (data from Camellato, et al. [2024]). Conversely, Puffin-D predicts no transcription in two reversed versions of the *HPRT1* sequence, consistent with experimental observations (Camellato, et al. 2024). Plotted are the number of GRO-cap reads observed experimentally or predicted by Puffin-D for the forward (native) *HPRT1* sequence, and predicted by Puffin-D for reversed and reversed-with-CpG-sites-removed *HPRT1* sequences, as indicated. Each point is the prediction for one base pair within each 100 kb sequence. Most nucleotides have no predicted transcription initiation. Puffin-D predicts transcription initiation in log10(s+1), where s = read number, thus the plots are Puffin-D values that were converted to read (pseudo-read) numbers.

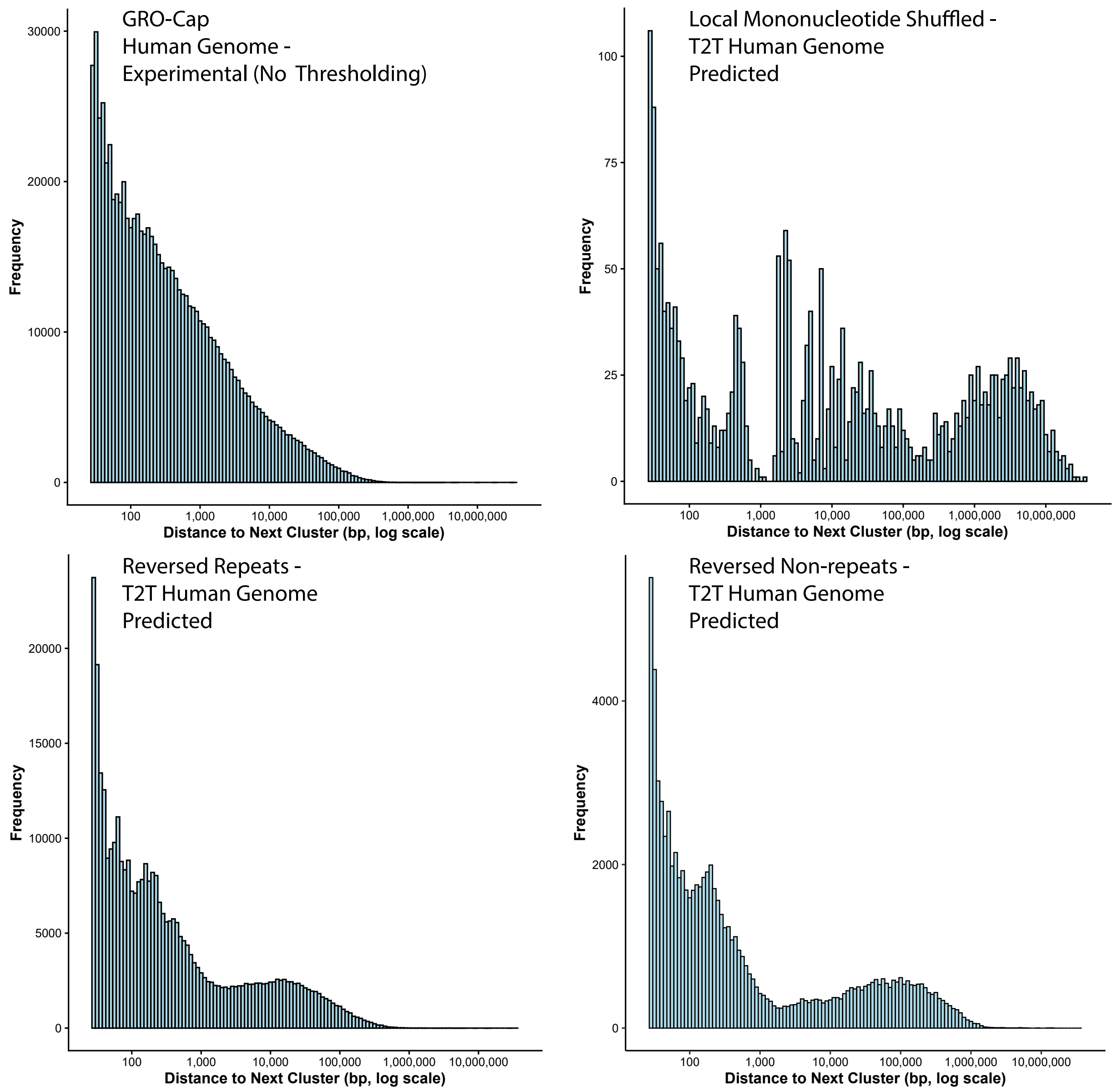

Figure S3. Trimodal distribution of inter-TSS cluster distances persists in different randomized genomes. Histograms of distances between TSS clusters from the data in **Table 1** are plotted for experimental GRO-Cap data without any read threshold, the local mononucleotide shuffled human genome, and versions of the human genome where just the repeat regions or just the non-repeat regions are reversed, as indicated. Inter-cluster distances are much longer (note the *x*-axis is log scale) as initiation frequency becomes sparser (indicated by the *y*-axis scale).

Table S1: Transcription initiation metrics as a function of different read number thresholds for the experimental GRO-cap dataset

Threshold (reads)[a]	Number of TSSs	Number of TSS clusters[b]	Mean cluster width[c]	Mean number of TSSs per cluster	Mean inter-TSS distance	Mean inter-TSS cluster distance
**1**	2,169,441	742,037	11.8	2.92	1,389	4,050
**2**	1,527,534	549,135	11.7	2.78	1,971	5,472
**3**	1,067,815	386,685	11.5	2.76	2,816	7,766
**4**	748,500	261,729	11.9	2.86	4,011	11,460
**5**	544,453	178,827	12.8	3.04	5,440	16,553
**6**	416,964	128,729	13.8	3.24	7,099	22,986
**7**	335,927	99,202	14.5	3.39	8,805	29,807
**8**	281,937	81,526	15.0	3.46	10,470	36,203
**9**	243,563	70,290	15.0	3.47	12,119	41,992
**10**	215,057	62,370	15.0	3.45	13,713	47,281
**11**	192,763	56,627	14.8	3.40	15,298	52,079
**12**	174,646	52,082	14.6	3.35	16,885	56,625
**13**	159,821	48,490	14.3	3.30	18,449	60,815
**14**	147,194	45,323	14.1	3.25	20,030	65,061
**15**	136,506	42,585	13.9	3.21	21,598	69,246

[a] The threshold number of reads required for a TSS to be called
[b] Clusters are groups of TSSs where the maximum distance between any two adjacent TSSs is 25 bp
[c] Average span of TSS clusters in bp
